# Use and User Experience of a Preconception Lifestyle App for Couples Undergoing in Vitro Fertilization: Mixed Methods Study

**DOI:** 10.2196/65815

**Published:** 2025-10-14

**Authors:** Tessy Boedt, Sharon Lie Fong, Eline Dancet, Merijn Mestdagh, Joke Verbeke, David Geerts, Carl Spiessens, Christophe Matthys

**Affiliations:** 1 Clinical and Experimental Endocrinology Department of Chronic Diseases and Metabolism KU Leuven Leuven Belgium; 2 Department of Developmental Psychology Tilburg School of Social and Behavioral Sciences Tilburg University Tilburg The Netherlands; 3 Department of Development and Regeneration KU Leuven Leuven Belgium; 4 Leuven University Fertility Center University Hospitals Leuven Leuven Belgium; 5 Department of Public Health and Primary Care KU Leuven Leuven Belgium; 6 Center for Psychology of Learning and Experimental Pyschopathology KU Leuven Leuven Belgium; 7 Health Intelligence Network Ghent (HINT.GENT) Faculty of Medicine and Health Sciences Ghent University Gent Belgium; 8 Institute of Media Studies KU Leuven Leuven Belgium; 9 DIEET Department of Endocrinology University Hospitals Leuven Leuven Belgium; 10 DIEET Department of Chronic Diseases and Metabolism KU Leuven Leuven Belgium; 11 Division of Human Nutrition Faculty of Medicine & Health Sciences Stellenbosch University Capetown South Africa

**Keywords:** mobile health, mHealth, infertility, lifestyle, usability

## Abstract

**Background:**

Mobile apps are a promising way to improve healthy lifestyle behavior among people with infertility. However, sufficient engagement with mobile health apps is crucial to influence health outcomes, and identifying features to create more effective interventions is urgently needed.

**Objective:**

This study conducted a process evaluation focusing on the use and user experience of the PreLiFe app, a mobile lifestyle app for couples undergoing in vitro fertilization (IVF).

**Methods:**

A mixed methods approach was used among heterosexual couples with infertility undergoing IVF. An objective quantitative study using a tracking-based system assessed the actual use of the PreLiFe app over time in relation to partner use and in relation to the specific fertility treatment. A subjective quantitative study using online questionnaires assessed the acceptability (using the Mobile App Rating Scale) and partner support (based on the Social Support for Diet and Exercise Scale) experienced while using the PreLiFe app. A subjective qualitative study using semistructured interviews evaluated in-depth user experiences with the PreLiFe app.

**Results:**

A total of 106 couples used the PreLiFe app for 2 to 365 days. Overall use was low; 18.9% (20/106) of the men and 49.1% (52/106) of the women used all the modules of the PreLiFe app. Mixed-model analyses revealed that higher app use was observed when a partner used the app as well and during fertility treatment. The average acceptability score was 6 (SD 1) of 10, and patients felt supported by their partners while using the app. Semistructured interviews with 10 patients indicated that the PreLiFe app was easy to use.

**Conclusions:**

Our findings showed good acceptability and user experiences but low actual objective use of a preconception lifestyle app for couples undergoing IVF. To increase use of and engagement with such apps, future studies should further focus on personalization and interaction with partners, health care providers, and other patient data systems.

## Introduction

### Background

Infertility, defined as the failure to achieve a clinical pregnancy after at least 12 months of regular unprotected sexual intercourse, occurs in 1 in 10 couples [1-3]. Approximately half of these couples undergo fertility treatment, such as in vitro fertilization (IVF), with the ultimate goal of conceiving a healthy child [3]. Infertility and its treatments can be emotionally and financially burdensome to couples and society. An important research priority is to find modifiable factors that can improve the chance of conceiving a healthy child from IVF [4]. Increasing evidence shows that lifestyle is such a potential modifiable factor. IVF success rates are positively associated with a healthy diet, a normal BMI, and moderate physical activity levels [5-12]. Furthermore, a healthy preconception lifestyle benefits offsprings’ health [13]. Several international guidelines recommend addressing unhealthy lifestyles before conception, specifically in people with infertility [14-16]. In contrast, a Cochrane review indicated a lack of clear guidance on the content and format of lifestyle programs for improving conception chances [17]. Health care providers also recognize the need for such guidance [18,19].

Evidence from other fields shows that mobile health (mHealth)—defined as the use of mobile and wireless technologies for health—is a promising format to promote healthy lifestyle behavior [20]. mHealth is widely scalable, relatively cheap, and adaptable according to the needs of patients [21]. Using mHealth among people with infertility may counter identified barriers such as lack of time and personnel and is easy to implement in daily life [20,22]. Recent studies on lifestyle apps for people with infertility have shown an improvement in secondary lifestyle outcomes (eg, food intake) but were not effective for the primary clinical outcomes, such as pregnancy rate or live birth [11,23-25]. Adherence is a challenge in traditional lifestyle studies. Therefore, in-depth analyses of use data of mHealth interventions is crucial to identifying factors that enhance use and engagement by participants and effectiveness of mobile preconception lifestyle interventions. In 2016, the World Health Organization mHealth Technical Evidence Review Group developed a guideline for the reporting of mHealth interventions. This guideline emphasized the need for detailed reporting on use [26]. Despite these existing guidelines, a review of mobile lifestyle interventions emphasized the need for improved reporting on the use of such interventions to evaluate their effectiveness more accurately [27].

### Objectives

To gain better insights into the effectiveness of a preconception lifestyle app for couples undergoing IVF (PreLiFe study) [22,25], a process evaluation focusing on use and user experiences was conducted with the aim of identifying factors that may lead to higher use and engagement, ultimately enhancing the effectiveness of mobile lifestyle apps.

## Methods

### Overview

This process evaluation (Table 1) of the PreLiFe app, a mobile lifestyle app for couples undergoing IVF, included stakeholder interviews, quantitative observation, and data monitoring. More specifically, an objective quantitative study (data monitoring) using a tracking-based system assessed the actual use of the PreLiFe app over time in relation to partner use and in relation to the specific fertility treatment. A subjective quantitative study using online questionnaires assessed acceptability and partner support experienced while using the PreLiFe app. A qualitative study (stakeholder interviews) using semistructured interviews evaluated the user experiences with the PreLiFe app in depth in the context of the users’ daily lives and in relation to their fertility treatment.

**Table 1:** Overview of outcomes assessed, timing, and measurement methods.

Outcomes assessed	Type of assessment	Timing of assessment	Method
Actual use of the PreLiFe app	Quantitative and objective	Continuously throughout the PreLiFe study period (2 to 365 days)	App-based tracking (data monitoring)
Acceptability—subjective quality	Quantitative and subjective	At the end of the PreLiFe study period	Subjective quality subscale of the Mobile Application Rating Scale
Acceptability—added value of different modules of the PreLiFe app	Quantitative and subjective	At the end of the PreLiFe study period	Semi–open-ended question
Acceptability—reasons for not using the PreLiFe app	Quantitative and subjective	After 3 months of using the PreLiFe app	Semi–open-ended question
Partner support experienced while using the PreLiFe app	Quantitative and subjective	After 3 months of using the PreLiFe app	Questionnaire based on the Social Support for Diet and Exercise Scale
User experience	Qualitative and subjective	After the PreLiFe study period	Semistructured interviews with stakeholders

### PreLiFe App and Study (Background Information)

The effectiveness of the PreLiFe program was evaluated in the PreLiFe study [25]. The protocol for this multicenter, single-blind randomized controlled trial has been registered (ClinicalTrials.gov identifier NCT03790449) and published previously [28]. A detailed description of the PreLiFe program has been published previously [22]. Briefly, eligible couples starting IVF were randomized (1:1 allocation ratio) to an attention control arm or an intervention arm receiving the PreLiFe program. The PreLiFe program is a systematically developed complex preconception lifestyle intervention acting on a combination of lifestyle topics (diet, physical activity, and emotional distress) and offered in a blended care format. It was developed according to the Medical Research Council framework, and patients were involved in the development of this app from the earliest phases of design [22]. The program includes a mobile app (PreLiFe app) with on-demand tailored advice and skill training on diet (food literacy), physical activity, and mindfulness in combination with SMS text messages. In addition, patients receive telephone interaction every 3 months with a health care provider trained in motivational interviewing (Figure S1 in Multimedia Appendix 1). Participants were followed for 12 months or until an ongoing pregnancy was confirmed via ultrasound at 12 weeks of gestational age. Changes in lifestyle and in reproductive outcomes were observed. The study started in January 2019 and was prematurely stopped on March 13, 2020, as, due to the COVID-19 pandemic, all Belgian fertility clinics were closed for an undefined period. As the primary outcome was time to ongoing pregnancy, recruitment was stopped as of March 13, 2020. In addition, couples who had been randomized and were still participating in the trial at that time needed to be censored. The PreLiFe study period varied from 2 to 365 days. Table S1 in Multimedia Appendix 1 provides an overview of the number of couples in the study at each time point and reasons for ending the PreLiFe study. During the PreLiFe study period, all participating couples received standard medical treatment (ie, IVF with or without intracytoplasmic sperm injection) according to the local protocol of the participating fertility clinic. Both partners in couples randomized to the intervention arm additionally received the PreLiFe program.

### Ethical Considerations

This study was approved by the Ethics Committee Research of the University Hospitals Leuven and the local ethics committees of the participating clinics (approval s61202 for the qualitative evaluation and s61596 for the quantitative evaluation). Written informed consent was obtained from all participants. Interviews were audio-recorded and transcribed verbatim. All identifying information was removed from the transcripts to ensure anonymity.

### Quantitative Evaluation: Actual Use, Acceptability, and Partner Support

#### Participants and Recruitment

As we aimed to evaluate the use of the PreLiFe app, only participants allocated to the intervention arm of the PreLiFe study were eligible for this study, which included 106 couples undergoing IVF.

#### Outcomes

Actual use of the PreLiFe app was objectively monitored through app-based tracking (data monitoring) throughout the PreLiFe study period. Initiation of the different modules (diet, physical activity, and mindfulness) was assessed by counting the number of times the modules were opened. Actual use of the different modules was assessed by automatically counting the active clicking on the features of these modules during the PreLiFe study period. Active use of the PreLiFe app was defined as the percentage of participants having used all modules (diet, physical activity, and mindfulness) of the PreLiFe app at least once throughout the PreLiFe study period.

Acceptability of the PreLiFe app was assessed at the end of the PreLiFe study period using the subjective quality subscale of the Mobile Application Rating Scale. This scale has a score from 0 to 10, with higher scores indicating better acceptability. Furthermore, we asked which module (diet, physical activity, or mindfulness) provided the most added value to participants and if there were any specific reasons (time, interest, use of other app or program, injury, or other reasons) for potentially not using the PreLiFe app. Multiple answers could be indicated.

To evaluate whether participants felt supported by their partners while using the PreLiFe app in maintaining a healthy lifestyle, a questionnaire based on the Social Support for Diet and Exercise Scale was developed [29]. The questionnaire consisted of 3 subscales: partner support for diet, physical activity, and emotional distress.

#### Statistical Analyses

Using mixed-model analyses, we evaluated the actual use of the different modules of the PreLiFe app over time and in relation to partner use. Furthermore, we evaluated the actual use of the PreLiFe app in relation to the stage of fertility treatment. Start of fertility treatment was defined as the start date of the last menstrual period. Descriptive analyses were conducted on the other outcomes (baseline characteristics, acceptability, added value, reasons for not adhering to the PreLiFe app, and partner support).

### Qualitative Evaluation: User Experiences

#### Participants and Recruitment

After the PreLiFe study was ended, participants from the intervention group were invited through email. The study process and accompanying incentives were explained in detail. Respondents who were interested in participating signed an online informed consent form and were scheduled for a semistructured interview through videoconferencing software (Microsoft Teams). Participants received a monetary incentive for taking part. The sample size was based on data saturation, with participants selected to ensure a diverse representation of age, gender, and app use, capturing a broad range of experiences.

#### Interview Guide and Process

Semistructured interviews were conducted by a researcher trained in qualitative methods according to an interview guide (Table S2 in Multimedia Appendix 1) and audio recorded for later analysis. The interview guide was developed from a user experience perspective grounded in the unified theory of acceptance and use of technology and behavior change theories [30]. It covered general app use, use and experiences of the different modules of the PreLiFe app (diet, physical activity, and mindfulness), integration into daily life and treatment, social influences, and technology acceptance. Questions were refined through pilot-testing with a small user sample (n=2) to ensure clarity, flow, and sensitivity.

#### Statistical Analyses

The data collected in the interviews were analyzed according to the principles of thematic analysis [31]. Interviews were audio recorded, transcribed afterward, and coded, after which themes were extracted through an affinity diagram.

## Results

### Quantitative Evaluation: Actual Use, Acceptability, and Partner Support

#### Baseline Characteristics of the Participants

The baseline characteristics of the participants are presented in Table 2. More than 70% of the couples were highly educated, and almost all couples were of European origin. The mean duration trying to conceive was approximately 2 years (27, SD 16 months).

**Table 2:** Baseline characteristics of the study participants.

Characteristic		Qualitative study			Quantitative study	
		Women (n=5)	Men (n=5)	Women (n=106)		Men (n=106)
Age (y), mean (SD)		30.2 (3.2)	32.6 (4.1)	30.7 (3.9)		33.9 (6.1)
**BMI^a^, n (%)**						
	Underweight (<18.5 kg/m^2^)	0 (0)	0 (0)	2 (1.9)		2 (1.9)
	Normal weight (18.5-24.99 kg/m^2^)	2 (40)	2 (40)	58 (54.7)		38 (35.8)
	Overweight (25-29.99 kg/m^2^)	2 (40)	3 (60)	30 (28.3)		52 (49.1)
	Obesity (≥30 kg/m^2^)	1 (20)	0 (0)	16 (15.1)		13 (12.3)
	Unknown	0 (0)	0 (0)	0 (0)		1 (0.9)
European origin^b^, n (%)		5 (100)	5 (100)	99 (93.4)		101 (95.3)
**Educational level^b^, n (%)**						
	ISCED^c^ level 0-3	0 (0)	0 (0)	20 (18.9)		34 (32.1)
	ISCED level 4-6	3 (60)	3 (60)	59 (55.7)		48 (45.3)
	ISCED level 7-8	2 (40)	2 (40)	27 (25.5)		24 (22.6)
Duration of time trying to conceive (months)^b^, mean (SD)		26 (14)	26 (14)	27 (16)		27 (16)
**Type of infertility^d^, n (%)**						
	Primary	4 (80)	4 (80)	79 (74.5)		78 (73.6)
	Secondary	1 (20)	1 (20)	27 (25.5)		28 (26.4)
**Female or male factor infertility diagnosis^d^, n (%)**						
	No	2 (40)	1 (20)	52 (49.1)		33 (31.1)
	Yes	3 (60)	4 (80)	52 (49.1)		71 (67)
	Unexplained	0 (0)	0 (0)	2 (1.9)		2 (1.9)

^a^Measured using weight and height.

^b^Self-reported.

^c^ISCED: International Standard Classification of Education.

^d^Obtained from medical records.

#### Actual Use of the PreLiFe App

From January 2019 to March 2020, a total of 106 couples used the PreLiFe app for 2 to 365 days. As shown in Table 3, actual use of the app was low. Active users, namely, participants who used every module of the app at least once, included 49.1% (52/106) of the women and 18.9% (20/106) of the men. Approximately 70% of the women (79/106, 74.5%) and 50% of the men (50/106, 47.2%) used at least one of the modules (diet, physical activity, or mindfulness). Furthermore, passive users, defined as participants who initiated the app without using any of the modules, included 9.4% (10/106) of the women and 37.7% (40/106) of the men. In total, 1.9% (2/106) of the women and 1.9% (2/106) of the men did not initiate the app at all (nonusers).

**Table 3:** Actual use of the PreLiFe app.

			Women (n=106)		Men (n=106)
**Diet**					
	Diet module initiated (questionnaire), n (%)	93 (87.7)		58 (54.7)	
	Diet module used (receiving tips and at least one goal set on food literacy), n (%)	79 (74.5)		38 (35.8)	
	>1 goal set, n (%)	36 (34)		11 (10.4)	
	Goals, range	1-7		1-7	
**PA^a^**					
	PA module initiated (questionnaire), n (%)	94 (88.7)		70 (66)	
	PA module used (receiving tips and at least one registration of step count or PA monitoring), n (%)	75 (70.8)		50 (47.2)	
	Step count function used, n (%)	71 (67)		48 (45.3)	
	Frequency of step count, range	8-200		8-242	
	Monitoring used, n (%)	31 (29.2)		23 (21.7)	
	Frequency of monitoring, range	1-236		1-138	
**Mindfulness**					
	Mindfulness module initiated (introductory movie), n (%)	76 (71.7)		41 (38.7)	
	Mindfulness module used (receiving tips and at least one exercise followed), n (%)	70 (66)		39 (36.8)	
	>1 exercise followed, n (%)	55 (51.9)		25 (23.6)	
	Frequency of exercising, range	1-19		1-22	
**Overall use, n (%)**					
	All modules initiated	68 (64.2)		32 (30.2)	
	All modules used (active users)	52 (49.1)		20 (18.9)	
	App initiated but no modules used (passive users)	10 (9.4)		40 (37.7)	

^a^PA: physical activity.

#### Actual Use of the Different Modules of the PreLiFe App Over Time in Relation to Partner Use

**Figure 1:**
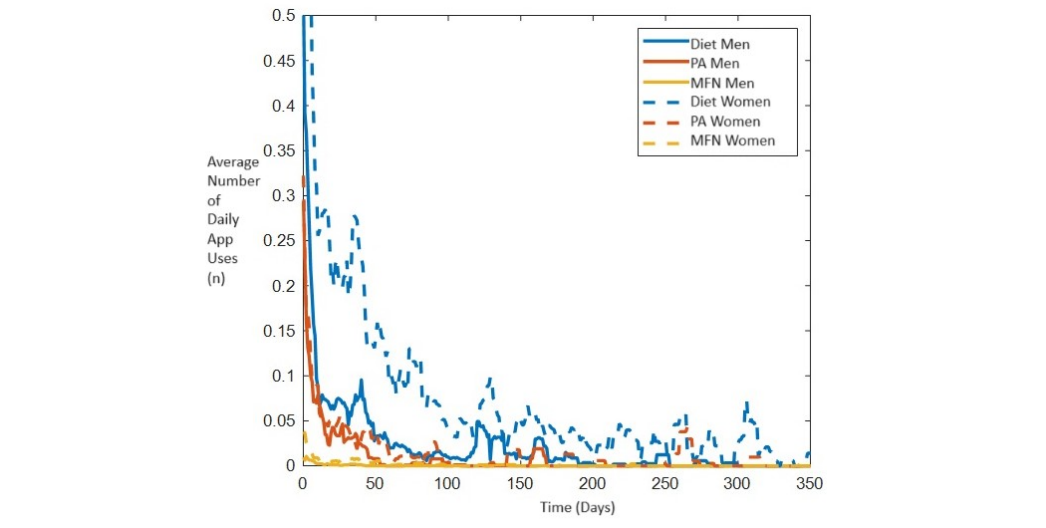
Use of the PreLiFe app over time. MFN: mindfulness; PA: physical activity.

Figure 1 presents an overview of the average number of daily app uses (diet, physical activity, and mindfulness) by both partners over time. Statistical analyses revealed the following: (1) the average number of daily app uses decreased over time; (2) women used the PreLiFe app more frequently as compared to their partners; and (3) on the days when one of the partners used the app, the other partner also used the app more (Table S3 in Multimedia Appendix 1).

The diet module was the most used. The average number of daily uses per person of the diet module was 0.2149 (SD 0.4636). The mindfulness module was the least used. The average number of daily uses per person was 0.005 (SD 0.0707).

#### Actual Use of the PreLiFe App in Relation to Fertility Treatment

**Figure 2:**
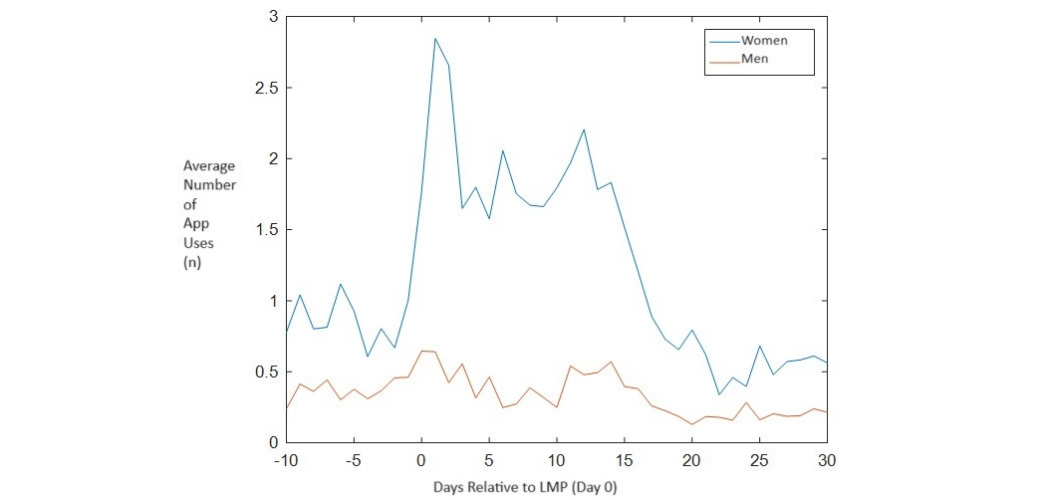
Use pattern of the PreLiFe app during in vitro fertilization (IVF) and intracytoplasmic sperm injection (ICSI). Day number relative to LMP refers to the day in the IVF and ICSI cycle that treatment was started (0=last menstrual period [LMP] day).

Figure 2 shows that the use of the PreLiFe app was highest around the first day of the menstrual period (ie, the start of ovarian stimulation in the couples’ first IVF–intracytoplasmic sperm injection treatment). We observed no differences in app use during the embryo transfer period. We did observe a decrease in app use across cycles (Figure S2 in Multimedia Appendix 1).

#### Acceptability of the PreLiFe App

A total of 40.6% (43/106) of the women and 33% (35/106) of the men completed the questions on acceptability of the PreLiFe app. Acceptability of the program revealed an average score of 6/10 (SD 1) from women and men, with a minimum score of 3/10 and a maximum score of 8/10. Women and men would recommend the PreLiFe app to other patients undergoing IVF (Table 4). Men rated the app slightly higher than women. In total, 26% (11/43) of the women gave a 4-star rating to the PreLiFe app versus 46% (16/35) of the men. Both women and men indicated that the diet module provided the most added value to them.

**Table 4:** Acceptability and added value of the PreLiFe app.

		Women (n=43), n (%)	Men (n=35), n (%)
“**Would you recommend the PreLiFe app to other patients undergoing IVF who might benefit from it?”**			
	Not at all	0 (0)	2 (6)
	To very few people	3 (7)	3 (9)
	To some people	26 (60)	17 (49)
	To many people	6 (14)	11 (31)
	To everyone	8 (19)	2 (6)
“**How would you rate the PreLiFe app?”**			
	1 star (“one of the worst apps I’ve used”)	0 (0)	0 (0)
	2 stars	2 (5)	4 (11)
	3 stars (average)	30 (70)	15 (43)
	4 stars	11 (26)	16 (46)
	5 stars (“one of the best apps I’ve used”)	0 (0)	0 (0)
**Module that provided the most added value**			
	Diet	19 (44)	15 (43)
	Physical activity	11 (26)	9 (26)
	Mindfulness	7 (16)	5 (14)
	No reply	6 (14)	6 (17)

A total of 26.4% (28/106) of the women and 22.6% (24/106) of the men completed the questions on reasons for not using the PreLiFe app. Table 5 provides an overview of these self-reported reasons. The most commonly indicated reason for not using the PreLiFe app was lack of time. In total, 14% (4/28) of the women also indicated that the PreLiFe app did not provide enough push messages.

**Table 5:** Reasons for not using the PreLiFe app.

Reasons	Women (n=28), n (%)	Men (n=24), n (%)
No time	16 (57)	18 (75)
Not interested	11 (40)	11 (46)
Use of other app or program	4 (14)	5 (21)
Injury or disease	1 (4)	0 (0)
Other	6 (21)	2 (8)
Limit on screen time	0 (0)	1 (4)
Not enough push messages	4 (14)	0 (0)
New phone	1 (4)	0 (0)
Failed fertility treatment	1 (4)	0 (0)

#### Partner Support Experienced While Using the PreLiFe App

A total of 44% (47/106) of the women and 33% (35/106) of the men completed the questions on partner support experienced while using the PreLiFe-app Regarding partner support experienced while using the PreLiFe app, more than half (30/47, 64%) of the women and men practiced a healthy diet often or very often together. One in 3 couples worked on their physical activity together. Furthermore, more than half (32/47, 68% and 25/35, 71%, respectively) of the women felt often supported by their partners in their diet. Only 0.9% (1/106) of the couples practiced mindfulness together. In addition, 38% (18/47) of the women and 46% (16/35) of the men never felt supported by their partners when practicing mindfulness (Table 6).

**Table 6:** Partner support experienced while using the PreLiFe app at 3 months.

			Women (n=47), n (%)							Men (n=35), n (%)				
			Diet		Physical activity		Mindfulness		Diet			Physical activity		Mindfulness
**Practiced with their partner**														
	Never	3 (6)		4 (9)		33 (70)		0 (0)			3 (9)		26 (74)	
	Seldom	2 (4)		9 (19)		5 (11)		2 (6)			9 (26)		4 (11)	
	A couple of times	10 (21)		15 (32)		3 (6)		8 (23)			8 (23)		3 (9)	
	Often	14 (30)		15 (32)		0 (0)		15 (43)			11 (31)		1 (3)	
	Very often	16 (34)		2 (4)		1 (2)		10 (29)			2 (6)		0 (0)	
	Not applicable	2 (4)		2 (4)		5 (11)		0 (0)			2 (6)		1 (3)	
**Felt supported by their partner**														
	Never	1 (2)		3 (6)		18 (38)		0 (0)			1 (3)		16 (46)	
	Seldom	3 (6)		7 (15)		5 (11)		1 (3)			7 (20)		4 (11)	
	A couple of times	10 (21)		11 (23)		6 (13)		5 (14)			6 (17)		3 (9)	
	Often	19 (40)		14 (30)		2 (4)		13 (37)			13 (37)		3 (9)	
	Very often	13 (28)		10 (21)		2 (4)		13 (37)			3 (9)		0 (0)	
	Not applicable	1 (2)		2 (4)		14 (30)		3 (9)			5 (14)		9 (26)	

### Qualitative Evaluation: User Experiences

#### Baseline Characteristics of the Participants

A total of 10 participants (4 couples and n=2, 20% individual participants) took part in 6 semistructured interviews between January 2021 and March 2021. The sample consisted of 50% (5/10) male individuals and 50% (5/10) female individuals who had all participated in the PreLiFe study and received access to the PreLiFe app for 1 year or until ongoing pregnancy occurred. The baseline characteristics of the participants are presented in Table 2.

#### User Experiences

A total of 112 codes were categorized into eight themes: (1) general evaluation, (2) pick-and-mix functionalities, (3) descriptions of app use, (4) app impact, (5) non–app-dependent behavior, (6) trust, (7) problems with the app, and (8) suggestions.

#### General Evaluation

All 10 participants mentioned that the PreLiFe app was easy to use and understand and that an app such as this one should be available by default as a part of fertility treatment. However, some added that it should not be mandatory.

#### Pick-and-Mix Functionalities

An obvious theme within each of the interviews was the on-demand or pick-and-mix approach toward modules and even functionalities within modules that participants used. Participants tended to pick the modules or functionalities that fit best in their lives. They often mentioned finding specific parts of the app useful:

The possibility to choose cooking advice suitable to your level of kitchen prowess.

Participant 1

Conversely, participants also mentioned that specific modules, especially mindfulness, were “not for them” (participants 2 and 5).

#### Descriptions of App Use

When asked about the use of the PreLiFe app in different social contexts, many participants (7/10, 70%) mentioned that they would use the app or suggestions from the app together with their partner to support each other in improving their lifestyle and prepare for the fertility treatment. A lot of the app use depended on the time available. Some participants (3/10, 30%) mentioned only using the PreLiFe app when they had some free time. Others mentioned that “the short tips received through notifications also work well when you don’t have much time at your disposal” (participant 6). In addition, the timing and events during IVF treatment could have an impact on people’s app use. Several participants (6/10, 60%) mentioned mainly using the app during key moments of the treatment:

We used the app around important doctor visits.

Participant 8

One participant mentioned completely pausing their use of the app as they had received disappointing news during their treatment, wanting to step away from the treatment for a little while. Some people (2/10, 20%), mostly men, did not end up using the entire app much or at all:

I have more of a supportive role in the process, the application might be more useful for women.

Participant 3

#### App Impact

Several participants (7/10, 70%) mentioned that the PreLiFe app made them aware that a healthy lifestyle was relevant to their fertility treatment. In addition, several participants (6/10, 60%) mentioned that using the PreLiFe app increased awareness of current behavior and triggered potential behavior change. However, it was mentioned that it was not always easy to adhere to the planned behavior. It was also mentioned that receiving notifications triggered further app use. Participants mentioned that, throughout their treatment, they were trying to find some sense of control over or influence on the results of the treatment. Some participants (4/10, 40%) said that the PreLiFe app helped them obtain this feeling of control.

#### Non–App-Dependent Behavior

Many participants (8/10, 80%) mentioned that they were already using physical activity trackers and, therefore, did not use those parts of the PreLiFe app as it was not compatible with their wearables.

#### Trust

The fact that the PreLiFe app was developed through the efforts of a university and hospital and operated by scientists and health care providers had a very positive effect on trust in the app. Participants were also willing to share personal information with the app.

#### Problems With App Use

One participant mentioned that having a pending update for the app caused her to stop receiving new information. Other experienced problems were with the pedometer, which stopped working or provided inconsistent results as compared to their own activity trackers.

#### Suggestions

Throughout the interviews, participants made several suggestions that could be added to the PreLiFe app. Participants stated that the functionality could become more automated by integrating current health applications or activity trackers. In addition, participants suggested that it would be interesting if health care professionals could have access to the recorded data, allowing for tailored feedback on their behavior. This aligns with the wish to have a more direct connection to health care providers by, for instance, being able to directly ask questions to the medical staff through the app.

Some participants (4/10, 40%) highlighted the importance of support throughout the emotional aspect of the fertility treatment, for instance, providing contact information for emotional support providers such as fertility psychologists. In addition, it was mentioned that it would be good to have some sort of “emotional guardian” who could step in when things became too much. Others suggested investing in features to improve the social aspect of the app, for instance, designing features that allow for sharing recorded data between partners as a way to help them stimulate and motivate each other. One participant felt that it would be interesting to have a forum attached in which different app users could share experiences and motivations with other people using the app.

## Discussion

### Principal Findings

This paper describes the quantitative and qualitative evaluation of the use and user experience of the PreLiFe app, as well as the app’s acceptability and the partner support experienced.

Overall, patients were positive about the PreLiFe app, often felt supported by their partners while using the app, and were willing to use it, as revealed by our quantitative and qualitative subjective evaluations. However, our objective use data revealed that actual app use was low, particularly among men. We observed that PreLiFe app use was related to partner app use and to the stage of fertility treatment (higher app use during the start of IVF treatment). Furthermore, we observed that the module that had the highest number of interactions and highest level of tailoring (the diet module) had the highest use.

### Implications and Comparison With Previous Work

One of the most remarkable findings of this study was the paradox between the high scores for acceptability and subjective use and the low actual objective use, highlighting the importance of objective measurement methods for app use. In the context of behavior change, these findings could be explained by the fact that the participants were in a phase of being aware of the health benefit (contemplation phase) but not yet having enough motivation or awareness to proceed to action for behavior change (in this case, regular app use and follow-up goals). Focusing on intrinsic motivation and possibly external motivation (intermediate follow-up by a physician or health care professional) could potentially have a synergistic effect. Compliance and acceptability do not reflect the actual use of lifestyle apps. Comparing our results with those of other studies remains difficult. A recent systematic review of lifestyle apps indicated substantial variability in intervention characteristics and inconsistent reporting of use outcomes [27]. Future research should work with core outcome sets on use to address this inconsistency in selecting and reporting of use outcomes [32]. Although previous research has shown that patients undergoing IVF need support in lifestyle modification and prefer a mobile app as a time-efficient format [22], the actual use of our PreLiFe app was low, especially among men. A possible reason may be found in a recent study by Robertson et al [33], who showed that patients undergoing IVF might prefer focusing on their treatment instead of other factors such as emotional management or their lifestyle. Another potential explanation for the low use among men is that we did not consider gender-specific differences when developing our app. Previous studies have shown that men may have different perceptions of lifestyle relevance to the fertility trajectory or different coping strategies to handle infertility [34,35]. Furthermore, lifestyle behavior and motivation to improve their lifestyle might differ between partners. Further characterizing the current needs of both partners and tailoring lifestyle apps to their personalized needs and motivations can contribute to the engagement of both partners in such interventions.

A recent systematic review identified several factors that positively influence adherence to lifestyle apps: personal support, content personalization, individualized push notifications, and user-friendly design [36]. In our study, we observed that PreLiFe app use was higher when the partner used the app as well and that patients felt supported by their partners. This is in line with previous studies showing that involving both partners in lifestyle interventions can facilitate mutual support, behavior change, and compliance at little additional cost [23,37]. A recent Dutch study providing tailored coaching on vegetable, fruit, and folic acid intake; smoking; and alcohol consumption in a blended approach to couples trying to conceive reported improved lifestyle behavior, which was more robust when both partners participated [38]. These findings suggest that partner-based interventions may offer a comparative advantage over individual-focused apps. Future studies on lifestyle apps for people with infertility should further consider this partner interaction.

PreLiFe app use also increased during fertility treatment, when participants were in contact with health care providers. This supports the idea that blended care models combining digital tools with professional support may enhance engagement. Determining the optimal balance between digital autonomy and health care provider interaction remains a key question [39].

Identifying the balance between time investment and personalization is also key for future research on this topic. Participants indicated lack of time as the most important reason for not using the PreLiFe app. In contrast, we observed the highest use for the diet module, which was the module with the highest level of time investment, interaction (2 push messages per week), and tailoring (personalized goals based on a questionnaire).

Another improvement area defined was the lack of interaction with other data systems such as wearables. Integration of wearables could enhance personalization and efficiency, as observed in other health apps [40]. However, further research on this topic in this specific population is warranted.

### Strengths and Limitations

The main strength of this study is that we combined qualitative and quantitative data on the use and user experiences of both partners, allowing for an in-depth understanding of participants’ use and experiences with a preconception lifestyle app. Furthermore, to our knowledge, we are the first to provide objective insights into the actual use of a lifestyle app among both partners. We did so using app-based tracking, an objective measure not prone to social desirability bias. In-depth analysis of such use data can contribute to identifying and improving factors that lead to higher use and engagement, ultimately enhancing the effectiveness of lifestyle apps [41,42].

The limitations of this study should also be considered. First, all participants were asked about their rationale for not using the PreLiFe app, but fewer than 1 in 3 women and men replied, potentially not reflecting the views of all nonusers. In general, self-selection of the participants might have played a role throughout the PreLiFe study. Our study population had a higher physical activity level and lower rates of overweight and obesity than the general Belgian population [43,44]. Selection bias could have been present in our study, resulting in participants with a high interest in lifestyle and health. Furthermore, most patients were of European origin and highly educated, limiting the generalizability of our results. Finally, to avoid burdening the participants, the final results of the qualitative study were not returned to them for validation. This decision could potentially lead to misinterpretations of their true thoughts.

### Conclusions

In conclusion, our findings show good acceptability and user experiences but low actual objective use of a preconception lifestyle app for couples undergoing IVF. Lifestyle apps can empower patients to feel more in control and supported regarding their reproductive health. However, to increase use and potential effectiveness, future research on lifestyle apps for people with infertility should further focus on the personalization of such apps to individual needs and on the interaction with partners, health care providers, and other patient data systems such as wearables.

## Appendix

Multimedia Appendix 1 Supplementary material.

## Abbreviations

IVF: in vitro fertilization
mHealth: mobile health
