# Supplementary material for: Use and User Experience of a Preconception Lifestyle App for Couples Undergoing in Vitro Fertilization: Mixed Methods Study
Source: JMIR Hum Factors. 2025 Oct 14;12:e65815. doi: 10.2196/65815 (PMC12569494; doi:10.2196/65815)

Supplementary material

Supplementary Table 1. Number of couples in the PreLiFe-study at each time point and reasons for ending PreLiFe-study

Months	In study	Out due to ongoing pregnancy	Out due to IVF discontinuation	Out due to Covid-19 stop
0	106	0	0	0
3	66	26	1	13
6	36	16	3	11
9	21	4	6	5
12	8	2	2	9

Supplementary Figure 1. Overview of PreLiFe-app (Adapted from Boedt et al. 2021 Human Reproduction)

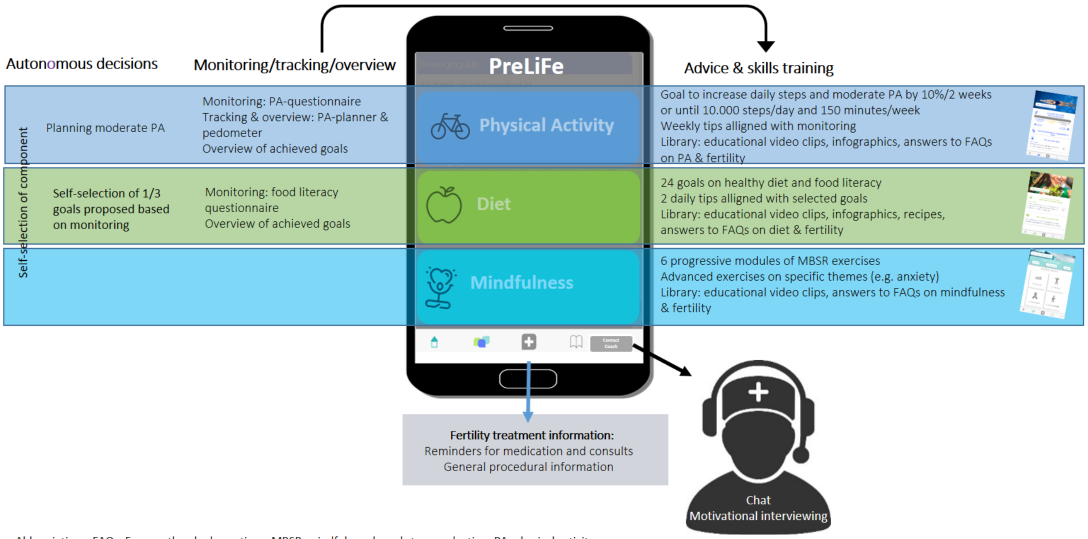

Abbreviations: FAQs: Frequently asked questions; MBSR: mindfulness based stress reduction; PA: physical activity

Supplementary Table 2. Interview guide of user experience research

Subject	Questions
Introduction<br>10-15min	Introduction Researcher:<br>- Background of researcher: User experience researcher, no health care professional<br>- Introduction about research aim<br>- Information about process (recording of interview)<br><br>Introduction Participants:<br>- Work<br>- Age<br>- Pregnancy status end of PreLiFe-study<br>- Fertility treatment<br>- App use after PreLiFe-study<br>- Anything else worth mentioning?
General experiences	Experiences with PreLiFe-app<br>Positive + example<br>Negative + example<br>Problems encountered?<br>How does the PreLiFe-app changes the experiences with fertility treatment
Modules	Use and experience of different modules:<br>- Diet<br>- Physical Activity<br>- Mindfulness<br>- Library<br>- Stepcount monitoring<br>- Textmessages with health care provider<br>- Coaching
People	Relation<br>- PreLiFe-app used together or alone?<br>- Influence of PreLiFe-app use on relationship?<br>Social environment<br>- Other persons that played a roll in PreLiFe-app use?
Places (location dependence of app usage)	Where did you use the PreLiFe-app?<br>Influence of location on PreLiFe-app use?
Routine (fit within daily routine)	How did the PreLiFe-app fit in your daily routine? Impact on daily routine?<br>How did the PreLiFe-app fit in your fertility treatment? Impact on treatment?
(Knowledge)	Diet<br>- Experience with diet module (self-knowledge)<br>- Influence on behavior? (awareness)<br>Physical activity<br>- Experience with physical activity module (self-knowledge)<br>- Experience with step count and physical activity registration<br>- Influence on behavior? (awareness)<br>Mindfulness<br>- Experience with mindfulness module (self-knowledge)<br>- Influence on behavior? (awareness)<br><br>Experience with data collection (reflection)<br>New insights?

Controle	Perceived sense of control on lifestyle advice?<br>PreLiFe-app use control on treatment?<br>Interaction with health care provider on PreLiFe-app use?
Technology Acceptance	PreLiFe-app useful?<br>Continue use of PreLiFe-app?<br>How difficult was it to use the PreLiFe-app?<br>Information sharing with PreLiFe-app?<br>Implementation of PreLiFe-app in standard of care?
End	What is currently missing in the PreLiFe-app, what would you improve?<br><br>Anything else to add? Any questions?

Supplementary Table 3. Mixed Model analyses to evaluate the actual use of the different modules of the PreLiFe-app over time, in relation to their partners' use and in relation to their stage of fertility treatment. Fixed effects coefficients (95% Confidence intervals). SE = Standard Error, DF = Degrees of Freedom LMP = Last menstrual period

Name	Estimate	SE	tstat	DF	p-value	Lower	Upper
Intercept	1.103	0.119	9.306	62955	1.368e-20	0.871	1.336
Female	0.406	0.085	4.794	62955	1.638e-06	0.240	0.572
Partner use	0.401	0.029	13.790	62955	3.399e-43	0.344	0.458
Days used	-0.011	0.001	-7.572	62955	3.723e-14	-0.014	-0.008
Important time:LMP	0.473	0.089	5.348	62955	8.923e-08	0.299	0.647

Supplementary Figure 2. Usage pattern of PreLiFe-app during IVF/ICSI per cycle. Day number relative to LMP, refers to the day in the IVF/ICSI cycle that treatment was started (LMP day = 0)

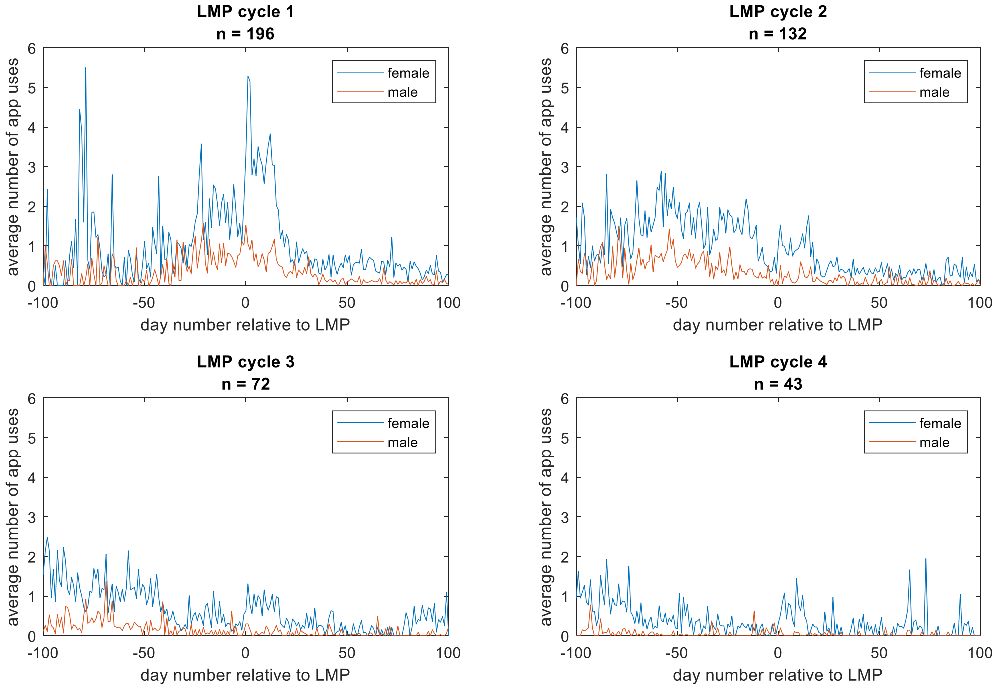

Supplement: Multimedia Appendix 1 [file humanfactors_v12i1e65815_app1.pdf]
